# Critical and non-critical coronavirus disease 2019 patients: which is the most predictive biomarker for disease severity and outcome? A multicentre prospective cohort study comparing mid-regional pro-adrenomedullin, inflammatory and immunological patterns

**DOI:** 10.1097/EA9.0000000000000039

**Published:** 2023-11-21

**Authors:** Giorgia Montrucchio, Eleonora Balzani, Gabriele Sales, Cesare Bolla, Cristina Sarda, Andrea Della Selva, Massimo Perotto, Fulvio Pomero, Enrico Ravera, Francesca Rumbolo, Tiziana Callegari, Vito Fanelli, Giulio Mengozzi, Luca Brazzi

**Affiliations:** From the Department of Surgical Sciences, University of Turin (GM, EB, GS, VF, lB), Department of Anaesthesia, Critical Care and Emergency – Città Della Salute e Della Scienza Hospital, Turin (GM, GS, VF, LB), Unit of Infectious Diseases, ASO SS. Antonio e Biagio e Cesare Arrigo, Alessandria (CB, CS), Department of Emergency, Anesthesia and Critical Care Medicine (ADS, ER), Department of Emergency Medicine (MP), Department of Internal Medicine, Michele e Pietro Ferrero Hospital, Verduno (FP), Clinical Biochemistry Laboratory, Città Della Salute e Della Scienza Hospital, Torino (FR, GM), Clinical Biochemistry Laboratory, ASO SS. Antonio e Biagio e Cesare Arrigo, Alessandria (TC) and Department of Medical Sciences, University of Turin, Torino, Italy (GM)

## Abstract

**BACKGROUND:**

Severe acute respiratory syndrome-coronavirus-2 in coronavirus disease 2019 (COVID-19) patients leads to a wide range of clinical manifestations. The evaluation of mid-regional pro-adrenomedullin (MR-proADM) as a prognostic biomarker in noncritical wards (NON-ICU) and intensive care units (ICU), may have a potential in predicting disease severity and outcomes.

**OBJECTIVE:**

To assess the difference in the prognostic power of MR-proADM in NON-ICU wards and in ICUs in a prospective multicentre cohort study.

**DESIGN:**

From January to July 2021, all adult COVID-19 patients requiring admission for more than 48 h.

**SETTING:**

One primary centre and two secondary centre hospitals.

**PATIENTS:**

One hundred and twenty-three ICU and 77 NON-ICU patients.

**INTERVENTION:**

MR-proADM, lymphocyte subpopulations and immunoglobulins were measured within 48 h and on days 3 and 7. A Log-rank test was used to compare survival curves, using a MR-proADM cut-off value of 1.5 nmol l^−1^. The predictive ability for mortality was compared using the area under the curve and 95% confidence interval (CI) of different receiver-operating characteristic curves.

**MAIN OUTCOME MEASURES:**

The first 48 h MR-proADM values were significantly higher in the ICU group (median value 1.10 [IQR, 0.80 to 1.73] pg ml^−1^ vs. 0.90 [0.70 to 1.20] pg ml^−1^, *P* = 0.020), and statistically significant changes were observed over time for MR-proADM, CD3+, CD4+ and CD56+. In univariate analysis, MR-proADM was the only biomarker that significantly predicted mortality (*P* = 0.006). The logistic regression model showed an odds ratio for mortality equal to 1.83 (95% CI, 1.08 to 3.37) *P* = 0.035 for MR-proADM, 1.37 (1.15 to 1.68) *P* = 0.001 for MuLBSTA and 1.11 (1.05 to 1.18) *P* less than 0.001 for SAPS II.

**CONCLUSION:**

MR-proADM admission values and trends over time appear to be a suitable marker of illness severity and a patient's risk of mortality in both ICU and NON-ICU settings. Lymphocyte subpopulation dysfunction seems to play a role in defining the severity of COVID-19 but is limited to ICU setting.

**TRIAL REGISTRATION:**

on clinicaltrials.gov, NCT04873388 registered on March 2020.

**KEY POINTS**

- In both ICU and NON-ICU COVID-19 patients, MR-proADM effectively predicts the severity of disease and predicts mortality, surpassing traditional biomarkers.
- Combining MR-proADM with clinical severity scores such as SAPS II and MuLBSTA improves its predictive value, aiding illness severity monitoring.
- T-lymphocyte dysfunction correlates with COVID-19 severity, especially in ICU patients, but further validation is needed.

## Introduction

Severe acute respiratory syndrome-coronavirus-2 (SARS-CoV-2) can cause a wide range of clinical manifestations, from mild respiratory dysfunction to severe complications such as acute respiratory distress syndrome (ARDS) and multiple organ failure.^1^ A dysregulated immune response is believed to contribute to the progression of coronavirus disease 2019 (COVID-19), as evidenced by alterations in various laboratory biomarkers such as lymphocyte counts and pro-inflammatory mediators.^2,3^

However, many of these biomarkers are not commonly used in clinical practice because of their poor analytical reliability, high cost and limited ability to distinguish between clinical conditions.^4,5^ In addition to cytokines, other biomarkers have shown promise in COVID-19 diagnosis, prognosis and in predicting outcomes. Studies have focused on comparing commonly used inflammatory markers [e.g. D-dimer, lactate dehydrogenase (LDH), C-reactive protein] with novel endothelial damage biomarkers and immunological markers (e.g. pro-adrenomedullin, lymphocyte subpopulations).^4–8^

Mid-regional pro-adrenomedullin (MR-proADM) is considered an effective biomarker of endothelial damage, and for that reason, it might be useful in conditions like septic shock, sepsis, pneumonia and ARDS.^9–12^ In the emergency department (ED) setting, MR-proADM has been compared with other severity scores with acceptable effectiveness in predicting the risk of mortality.^13^

As endothelitis has emerged as a cornerstone of SARS-CoV2 severity, an association between MR-proADM levels and endothelial damage caused by the virus has been hypothesised.^10,14^

Studies on MR-proADM as a prognostic biomarker in COVID-19 patients have shown heterogeneity in terms of populations, methods and findings.^15^ Most available studies have focused on analysis of a single value on admission to the emergency department^13^ or exclusively on patients in an intensive care unit (ICU).^16,17^ In this specific context, the role of repeated measurements is becoming increasingly important but there is a lack of studies comparing trends in inflammation biomarkers among COVID-19 patients with different clinical severities, such as NON-ICU vs. ICU patients.^18^ The role of immunological biomarkers and the depletion of leukocytes, immunoglobulins (Ig) and lymphocyte subpopulations in the context of viral infections has also been poorly studied.^19^

The aim of this prospective multicentre cohort study was to compare the prognostic power of MR-proADM in noncritical wards and ICU. Considering the association of MR-proADM with traditional clinical severity scores, this study aims to evaluate the effectiveness of MR-proADM in predicting disease severity and outcomes in COVID-19 patients. Additionally, the study aims to assess the correlation between lymphocyte subpopulations and Ig values with different levels of disease severity and their impact on patient outcomes.

## Methods

### Study design and setting

This observational prospective cohort study was conducted between January 2021 and July 2021. The study adhered to the principles outlined in the Declaration of Helsinki regarding biomedical research involving human subjects. The study's reporting followed the guidelines set by the STROBE (STrengthening the Reporting of OBservational studies in Epidemiology) checklist.^20^ Each participating centre obtained approval from their respective Institutional Review Board (IRB), and the study protocol was registered prospectively on clinicaltrials.gov (NCT04873388).

The study was carried out in three Italian centres: ’Città della Salute e della Scienza’ hospital in Turin, ’Michele e Pietro Ferrero’ hospital in Verduno and ’Azienda Ospedaliera Nazionale SS. Antonio and Biagio and C. Arrigo’ in Alessandria. The consort diagram is available in the supplementary materials (Supplementary Figure 1).

### Participants

All consecutive adult patients with coronavirus 2 (SARS-CoV-2) pneumonia, confirmed by the real-time PCR (RT-PCR) on at least one low respiratory tract specimen,^21^ and requiring hospital admission expected to last longer than 48 h were included. The patients were divided in two groups, according to their clinical severity [high severity patients in ICU and low severity patients in medical and infectious diseases wards (NON-ICU)]. Gravity scores, such as Sequential Organ Failure Assessment (SOFA), Simplified Acute Physiology Score II (SAPS II) and a score predicting viral pneumonia mortality (MuLBSTA)^22–24^ were used.

### Variables

#### Laboratory data and imaging

On admission (ICU or NON-ICU), all patients underwent clinical and routine laboratory tests assessment (including leukocytes and lymphocytes) in addition to inflammatory and fibrinolysis biomarkers such as C-reactive protein (CRP), procalcitonin (PCT), D-dimer, lactate dehydrogenase (LDH), N-terminal prohormone brain natriuretic peptide (NT-pro-BNP), performed according to the methods currently used in our laboratory. All these blood tests, together with MR-proADM, were measured within 48 h from ward admission (subsequently named as ‘predictive values’, T pred) and repeated at day 3 (after 72 h, T3) and at day 7 (T7). In addition, lymphocyte subpopulation [CD45+, CD3+, CD3+CD4+ (Th cells), CD3+CD8+, CD4+/CD8+, CD19+ (B lymphocytes), and CD16+CD56+ (NK cells)] count was analysed within 48 h from ward admission and on day 7.

MR-proADM concentrations were determined by the B.R.A.H.M.S. KRYPTOR compact PLUS (Thermo Fisher Scientific, Hennigsdorf, Germany) automated method using the TRACE (Time-Resolved Amplified Cryptate Emission) technique. The detection limit of this assay is 0.05 nmol l^−1^ while intra-assay and inter-assay coefficients of variation were less than 4% and less than 11%, respectively.

Lymphocyte immunophenotyping was performed by an AQUIOS CL Flow Cytometry System using two separate combinations of four or five murine monoclonal antibody panels, each conjugated to a specific fluorochrome and specific for a different cell surface antigen (Kits Tetra- Panels 1 and 2), as per the manufacturer's instructions (Beckman Coulter, Inc., Brea, California, USA).

Microbiological cultures of blood, bronchial aspirate or broncho-alveolar samples; and radiological investigation, chest X-rays or CT scans, were performed as clinically indicated.

The risk of bias was evaluated using The Risk Of Bias In Nonrandomised Studies – of Interventions (ROBINS-I) assessment tool.^25^

### Study size

The sample size for the study was calculated using the G^∗^Power 3.1 calculator (© 2023 Heinrich-Heine-Universität Düsseldorf). Based on the data previously collected at the principal centre, the researchers estimated a total population sample of 140 patients. With a desired α level of 0.05 and a power of 0.80, and allowing for a 15% dropout rate, the minimum estimated sample size required for the study was determined to be 180 patients but with a possibility to extend this because of the observational nature of the study, according to the study period.

### Statistical methods

Descriptive summary variables, in the general population and divided into ICU and NON-ICU patients, were presented as mean ± SD or median [IQR] for continuous variables and as percentages for categorical variables.

Shapiro normality test and one-way analysis of variance for continuous and categorical variables were performed. The analysis was then repeated between survivors and nonsurvivors.

For survival analysis, we used the Kaplan–Meier method considering a period of 28 days.

Trends with time (Tpred defined as the value measured within 48 h to the admission, T3 at day 3 and T7 at day 7) were evaluated with a multivariate analysis of variance.

A logistic regression model evaluating the correlation of mortality and MR-proADM predictive values adjusted for ward, age, previous smoking history, neutrophils, lymphocytes, neutrophil/lymphocyte ratio, T-lymphocyte overall values, T-lymphocyte subpopulations (CD3+CD4+, CD3+CD8+), CD56+ natural killer lymphocytes, MR-proADM, SAPS II, SOFA and MuLBSTA scores was performed. Results are presented as odds ratio (OR) and 95% confidence interval (CI). The logistic regression was repeated evaluating the impact of the single score of critical illness (SOFA, SAPS II) and potential superinfection (MuLBSTA). For each model, we assessed the area under the curve (AUC) of different receiver-operating characteristics curves (ROC) (Supplementary Figure 2). Accuracy, sensitivity, specificity, positive-predictive value (PPV) and negative-predictive values (NPV) were then assessed. For each model, the Akaike information criterion (AIC) was calculated. All tests were two-sided, and the level of statistical significance was set at 95% CI.

We used a random forest-based model of baseline characteristics to develop a classifier for predicting the ward where the patient should be admitted. The Random Forest model was created using the ’randomForest’ package, with the number of trees (ntree) set to achieve good predictive accuracy. Additionally, the proximity matrix, which represents the similarity between observations in the Random Forest model, was computed. To evaluate the model's performance, an Out-of-Bag (OOB) error analysis was conducted. This allowed for assessing the model's effectiveness in the context of new observations and obtaining an objective evaluation of its performance. To further optimise the model, an analysis was carried out to determine the optimal number of variables to use, and iterations with different combinations of predictive variables were performed.

Lastly, a two-dimensional graphical visualisation of the observations was created using the multidimensional scaling (MDS) technique. This enabled the visual representation of the relationships between observations in the dataset, highlighting any clusters or distinctive patterns.

All the analyses and graphs were performed with the open-source RStudio 2022.07.1.^26,27^

## Results

### Participants and descriptive data

Two hundred patients were enrolled, 123 patients in the ICU group and 77 in the NON-ICU group (Table 1). The mean ± SD age of the overall population was 65.3 ± 11.7 years, with a statistically significant difference between the ICU and the NON-ICU population: 63.9 ± 10.7 vs. 67.6 ± 12.8 years, respectively, *P* = 0.033).

**Table 1:** Descriptive data at baseline and during hospitalisation, disease severity scores and outcomes

Descriptive data	Overall (*n* = 200)	ICU (*n* = 123)	NON-ICU (*n* = 77)	*P*
Characteristics				
Age (years)	65.32 ± 11.68	63.87 ± 10.72	67.62 ± 12.82	**0.033**
BMI (kg m^−2^)	28 [25 to 31]	29 [25 to 32]	26 [24 to 28]	**<0.001**
Sex, male	134 (67)	88 (77.5)	46 (59.7)	0.084
Active smoking	19 (9.6)	18 (14.9)	1 (1.3)	**0.002**
Previous smoking history	46 (23.1)	25 (20.5)	21 (27.3)	0.269
Drug/alcohol	5 (2.5)	4 (3.3)	1 (1.3)	0.389
Cardiovascular disease	35 (17.5)	17 (13.8)	18 (23.4)	0.084
Patient underwent NIV	88 (44.0)	87 (70.7)	1 (1.3)	**<0.001**
Patient underwent IMV	120 (60.0)	110 (89.4)	10 (13.0)	**<0.001**
Neuromuscular block	108 (54.0)	107 (87.0)	1 (1.3)	**<0.001**
Prone positioning	121 (60.5)	99 (80.5)	22 (28.6)	**<0.001**
iNO	18 (9.0)	17 (13.8)	1 (1.3)	**<0.001**
ECMO during stay	13 (6.5)	13 (10.6)	0 (0.0)	**0.003**
RRT during stay	13 (6.5)	12 (9.8)	1 (1.3)	**0.01761**
Vasopressors during stay	92 (46.0)	90 (73.2)	2 (2.6)	**<0.001**
Disease severity scores				
SOFA score, at admission	4 [3 to 7]	6 [4 to 8]	2 [2 to 3]	**<0.001**
MuLBSTA score, at admission	11 [9 to 15]	13 [9 to 15]	11 [7 to 13]	**<0.001**
SAPS II score, at admission	34 [22 to 47]	42 [34 to 54]	21 [15 to 24]	**<0.001**
Outcomes				
Hospital length of stay (days)	20 [13.0 to 30.0]	23 [16.0 to 32.0]	16 [8.5 to 22.5]	**<0.001**
In hospital mortality	74 (37)	68 (56.7)	6 (7.8)	**<0.001**
Mortality in the ward	68 (34)	63 (51.2)	5 (6.5)	**<0.001**

Data are presented as mean ± SD, median [IQR] and *n* (%). BMI, body mass index; iNO, inhaled nitric oxide; MR-proADM, midregional-proadrenomedullin; NIV, noninvasive ventilation; SAPS II, Simplified Acute Physiology Score II; SOFA, Sequential Organ Failure Assessment.

On univariate analysis, the two patient groups were found to differ for BMI (*P* < 0.001), SOFA score (*P* < 0.001), MuLBSTA score (*P* < 0.001), SAPS II score (*P* < 0.001), pH at admission (*P* < 0.001) and lactate at admission (*P* < 0.001). Among comorbidities, only active smoking was found to be statistically different between groups (*P* = 0.002).

### Therapeutic approaches

Considering ICU vs. NON-ICU patients, oxygen support was required in 97.4 and 78.9% and high-flow nasal cannulae (HFNC) were used for 39.8 and 44.2%. Continuous positive airway pressure (CPAP) was used in 85.4 vs. 59.7%, and noninvasive ventilation (NIV) in 70.7 vs. 1.3%. Rescue ventilatory therapy (prone positioning) was used in 80.5 vs. 28.6% and inhalation of nitric oxygen (iNO) was used and in 13.8 vs. 1.3%. During the admission, renal replacement therapy (RRT) was required 9.8 vs. 1.3% and vasopressors in 73.2 vs. 2.6% (Table 1).

### Biomarkers and outcomes

MR-proADM, obtained within 48 h of admission, showed an overall median [IQR] value of 1.00 [0.80 to 1.50] pg ml^−1^. There was a statistically significant difference between the ICU patients 1.10 [0.80 to 1.73] pg ml^−1^ and the NON-ICU patients 0.90 [0.70 to 1.20] pg ml^−1^ (*P* = 0.020) (Table 2a). Statistically significant differences between the ICU and NON-ICU groups were observed for PCT, neutrophils, lymphocytes, neutrophil/lymphocyte ratio, overall T-lymphocyte populations and CD56 natural killer lymphocyte values (Table 2a).

**Table 2:** Predictive values in the general population and categorised according to the ward (ICU or NON-ICU) and within each ward between survivors and nonsurvivors

	Ward			
A. Predictive values^a^	Overall (*n* = 200)	ICU (*n* = 123)	NON-ICU (*n* = 77)	*P*
MR-proADM (pg ml^−1^)	1.00 [0.80 to 1.50]	1.10 [0.80 to 1.73]	0.90 [0.70 to 1.20]	**0.020**
CRP (mg l^−1^)	78 [34 to 137]	87 [36 to 151]	62 [30 to 129]	0.136
PCT (ng ml^−1^)	0.18 [0.08 to 0.74]	0.23 [0.10 to 0.73]	0.10 [0.05 to 1.09]	**0.007**
Neutrophils (cells μl^−1^)	7,515 [4,830 to 10,970]	9,420 [5,460 to 12,680]	6,070 [4,480 to 7,980]	**<0.001**
Lymphocytes (cells μl^−1^)	620 [420 to 905]	530 [360 to 758]	770 [560 to 1,050]	**<0.001**
Neutrophil/lymphocytes ratio	13 [7 to 22]	16 [9 to 26]	9 [5 to13]	**<0.001**
IgM (mg dl^−1^)	61 [1 to 108]	62 [2 to 109]	58 [1 to 106]	0.851
IgG (mg dl^−1^)	745 [11 to 961]	745 [12 to 938]	755 [11 to 977]	0.878
IgA (mg dl^−1^)	165 [3 to 251]	171 [4 to 271]	164 [3 to 232]	0.409
T lymphocytes [CD3+] (cells μl^−1^)	376 [216 to 577]	298 [189 to 455]	486 [359 to 761]	**<0.001**
T lymphocytes CD4 [CD3+CD4+] (cell μl^−1^)	259 [138 to 404]	199 [122 to 323]	341 [211 to 486]	**<0.001**
T lymphocytes CD8 [CD3+CD8+] (cells μl^−1^)	106 [60 to 154]	86 [44 to 114]	145 [90 to 260]	**<0.001**
B lymphocytes [CD19] (cells μl^−1^)	113 [66 to 180]	99 [57 to 178]	120 [71 to 182]	0.063
Natural killer [CD56+] (cells μl^−1^)	66 [33 to 117]	41 [20 to 69]	116 [73 to 224]	**<0.001**

	*P* values among survivors vs. nonsurvivors within each ward		
B. Predictive values^a^	Overall [*P*]	ICU [*P*]	NON-ICU [*P*]
MR-proADM (pg ml^−1^)	**0.002**	**<0.001**	**0.006**
CRP (mg l^−1^)	0.125	0.643	0.145
PCT (pg ml^−1^)	0.998	0.721	0.280
IgM (mg dl^−1^)	0.064	0.061	0.451
IgG (mg dl^−1^)	0.238	0.067	0.438
IgA (mg dl^−1^)	**0.050**	**0.016**	0.190
T lymphocytes [CD3+] (cells μl^−1^)	**0.011**	**0.030**	0.111
T lymphocytes CD4 [CD3+CD4+] (cells μl^−1^)	**0.021**	**<0.001**	0.104
T lymphocytes CD8 [CD3+CD8+] (cells μl^−1^)	0.061	0.091	0.334
B lymphocytes CD19+ (cells μl^−1^)	**0.003**	0.098	**0.025**
Natural killer CD56+ (cells μl^−1^)	**0.001**	**0.050**	0.774

Subpart A: biomarkers, lymphocytes subpopulations and Immunoglobulin type values at admission. Subpart B: univariate significance, relating to the values of biomarkers, lymphocytes subpopulations and immunoglobulin type, at admission, on the whole population and divided by ward (ICU vs. NON-ICU). Data are presented as median [IQR], if not otherwise specified. BMI, body mass index; iNO, inhaled nitric oxide; MR-proADM, midregional-proadrenomedullin; NIV, noninvasive ventilation; SAPS II, Simplified Acute Physiology Score II; SOFA, Sequential Organ Failure Assessment.

a Predictive values are assessed as the first value available within 48 h.

**Fig. 1:**
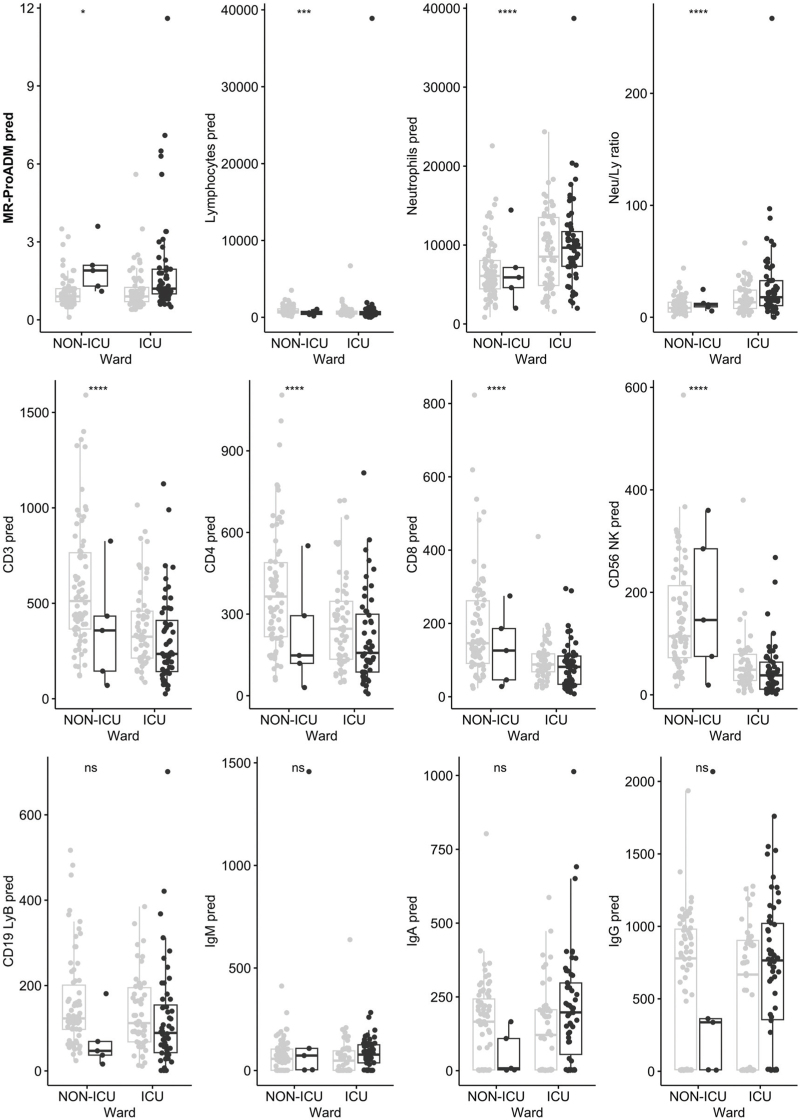
Box plots representing predictive values (day 0, day 1 and day 2) of biomarkers and lymphocyte subpopulation as indicated next to each graph.

The biomarker analysis between survivors and nonsurvivors is shown in Fig. 1. MR-proADM was the only biomarker that significantly predicted survival in the univariate analysis (*P* = 0.006) (Table 2b). In fact, when assessing the predictive ability of other biomarkers, T lymphocytes (*P* = 0.03), CD4 T lymphocytes (*P* < 0.001), CD56 NK lymphocytes (*P* = 0.05) and type A immunoglobulin (*P* = 0.016) reached significance only in the ICU subgroup, whereas only B lymphocytes reached significance in the NON-ICU subgroup (*P* = 0.025) (Table 2b).

Values are split by Ward (ICU and NON-ICU) and by mortality (grey, survivors; black, nonsurvivors). All individual data points are shown. The median is shown as the thick horizontal line across the box; the lower and upper limits of the box represent the 25th And 75th percentiles, respectively; the ends of the whiskers indicate 1.5^∗^IQR from either end of the box. Wilcoxon rank sum test and the respective *P* value were performed between wards. Codes for these *I* values are shown at the top right of individual boxplots: ns, not significant; ^∗^ = 0.05; ^∗∗∗^ = 0.01; ^∗∗∗∗^ = 0.001.

**Fig. 2:**
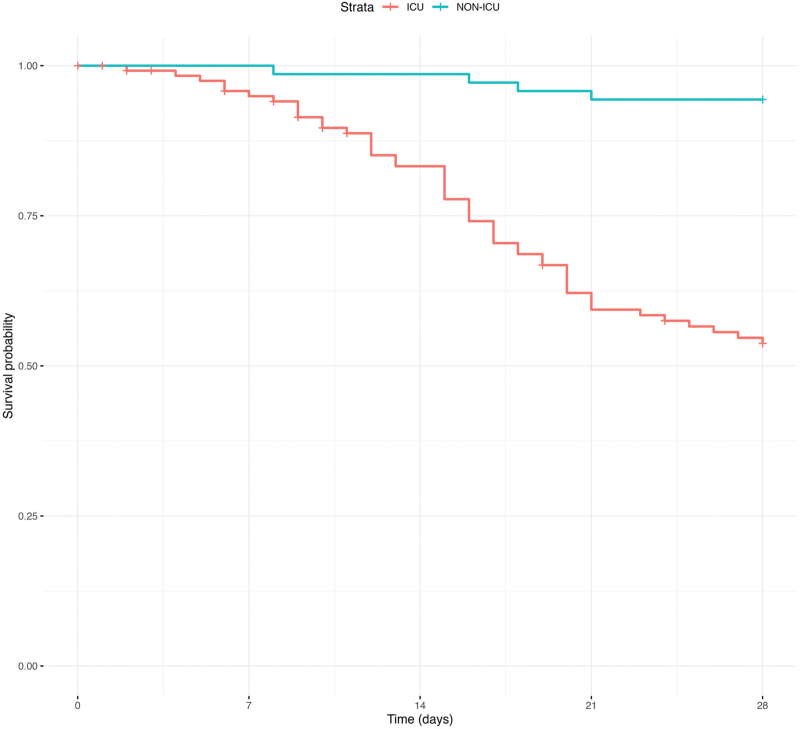
Kaplan–Meier survival curve for patients in ICU and NON-ICU ward.

Mortality for the entire cohort was 34% in the recruitment ward and 37% considering the total duration of their hospital stay. In the NON-ICU group, mortality was 6.5% in the ward and 7.8% while in the hospital, and for the ICU patients, mortality was 51.2% in ICU and 55.3% while in the hospital. The results of the univariate comparison between survivors and nonsurvivors in both the ICU and NON-ICU groups are shown in Table 1S. Overall 28-day mortality was analysed using a Kaplan–Meier curve with a Log-rank test (*P* < 0.010) (Fig. 2).

The graph shows the probability of survival over time, and the *y*-axis indicating the time in days. The red line represents in ICU, while the blue line represents patients in NON-ICU (log-rank *P* value <0.001).

### Logistic regression model, and forest plot analysis

The logistic regression model applied to test the impact of MR-proADM on mortality adjusted for ward, age, previous smoking history, total T-cell values, neutrophils, lymphocytes, neutrophil/lymphocyte ratio, T-cell subpopulations (CD3+ CD4+, CD3+CD8+), CD56+ natural killer lymphocytes, SAPS II, SOFA and MuLBSTA scores evidenced a statistically significant role for MR-proADM 1.83 [95% CI, 1.08 to 3.37) *P* = 0.035], MuLBSTA 1.37 (1.15 to 1.68) *P* = 0.001, and SAPS II, 1.11 (1.05 to 1.18), *P* < 0.001 (Table 3) with an estimated AIC and AUC of 133.1 and 0.922, respectively (Table 4, Supplementary Fig. 2) and an accuracy of 0.86 (95% CI, 0.80 to 0.91] for the model. Sensitivity, specificity, PPV and NPV of the model are shown in Table 4.

**Table 3:** Odds ratio values calculated on logistic regression

Value	OR (95% CI)	*P*
MR-proADM	1.83 (1.082 to 3.366)	0.026
SOFA	1.01 (0.772 to 1.327)	0.678
SAPS II	1.11 (1.051 to 1.178)	0.001
MuLBSTA	1.34 (1.152 to 1.676)	0.838
Age	0.98 (0.927 to 1.028)	0.493
Ward	1.18 (0.155 to 8.442)	0.001
Previous smoking history	3.64 (0.953 to 14.758)	0.173
Neutrophils	1.00 (1.000 to 1.000)	0.223
Lymphocytes	1.00 (1.000 to 1.001)	0.141
Neutrophil/lymphocyte ratio	1.01 (0.978 to 1.067)	0.193
T lymphocytes CD3+	1.02 (1.002 to 1.052)	0.982
T lymphocytes CD4 (CD3+CD4+)	0.98 (0.948 to 0.997)	0.026
T lymphocytes CD8 (CD3+CD8+)	0.98 (0.942 to 0.999)	0.678
Natural killer CD56+	1.00 (0.994 to1.009)	0.001

CI, confidence interval; MR-proADM, mid-regional pro-adrenomedullin; OR, odds ratio; SAPS II, Simplified Acute Physiology Score II; SOFA, Sequential Organ Failure Assessment.

**Table 4:** Predictive models based on multivariate analysis and their corresponding receiver-operating characteristics curve

	Accuracy (95% CI)	Sensitivity	Specificity	PPV	NPV	AUC	AIC
MR-proADM	0.79 (0.726 to 0.852)	0.907	0.539	0.817	0.718	0.880	160
SOFA	0.84 (0.77 to 0.890)	0.926	0.635	0.855	0.786	0.877	157.9
SAPS II	0.86 (0.802 to 0.910)	0.942	0.679	0.870	0.837	0.885	151.5
MuLBSTA	0.81 (0.746 to 0.868)	0.908	0.596	0.837	0.738	0.867	162.29
Logistic regression overall model	0.86 (0.799 to 0.910)	0.914	0.740	0.891	0.787	0.922	133.1

Adjusted for ward, age, previous smoking history, neutrophils, lymphocytes, neutrophil/lymphocyte ratio, T-lymphocyte overall values, T-lymphocyte subpopulations (CD3+CD4+, CD3+CD8+), CD56+ natural killer lymphocytes, SAPS II, SOFA, MR-proADM and MuLBSTA scores. AIC, Akaike information criterion; AUC, area under the curve; MR-proADM, midregional-proadrenomedullin; NPV, negative-predictive value; PPV, positive-predictive value; SAPS II, simplified acute physiology score II; SOFA, sequential organ failure assessment.

**Fig. 3:**
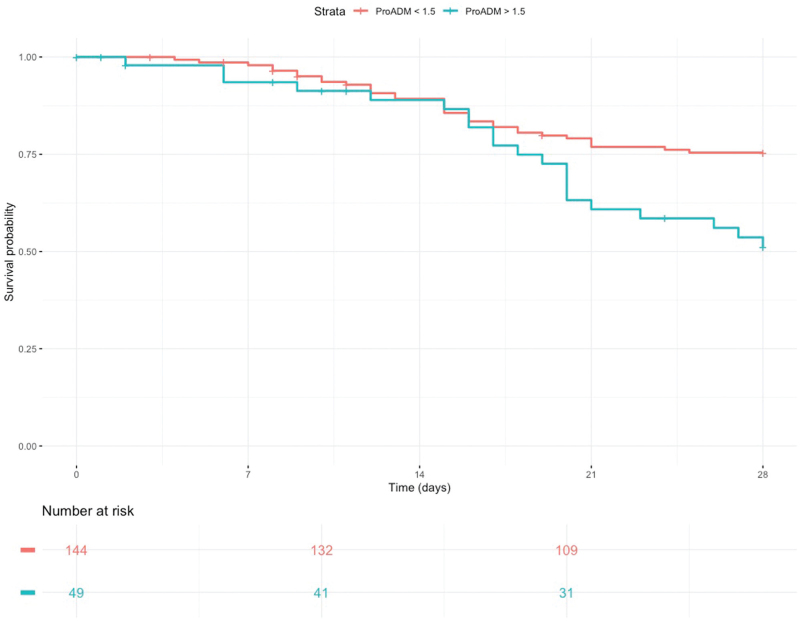
Kaplan–Meier survival curve for patients hospitalized in ICU and NON-ICU included in our study and divided by MR-proADM levels according to the literature cut-offs.

We conducted further multivariate analyses fitting logistic regression models for MR-ProADM, SOFA, MuLBSTA and SAPS II-predictive values and computed AIC, AUC, accuracy, sensitivity, specificity, PPV and NPV for each model (Table 4 and Supplementary, Figure 2). We stratify our population on the basis of the cut-off reported in the literature to define the risk of mortality in COVID = 19 patients (1.5 pg ml^−1^).^28^ We calculated the corresponding Kaplan–Meier curve and performed its log-rank test for 28-day mortality (Fig. 3).

The graph shows the probability of survival over time, with the *x*-axis indicating time in days and the *y*-axis indicating the proportion of patients who are alive. The blue line represents patients with MR-proADM levels below the cutoff of 1.5, while the yellow line represents patients with MR-proADM levels above the cutoff (log-rank *P*-value = 0.005) 0.88, indicating good discriminatory power of the mortality.

**Fig. 4:**
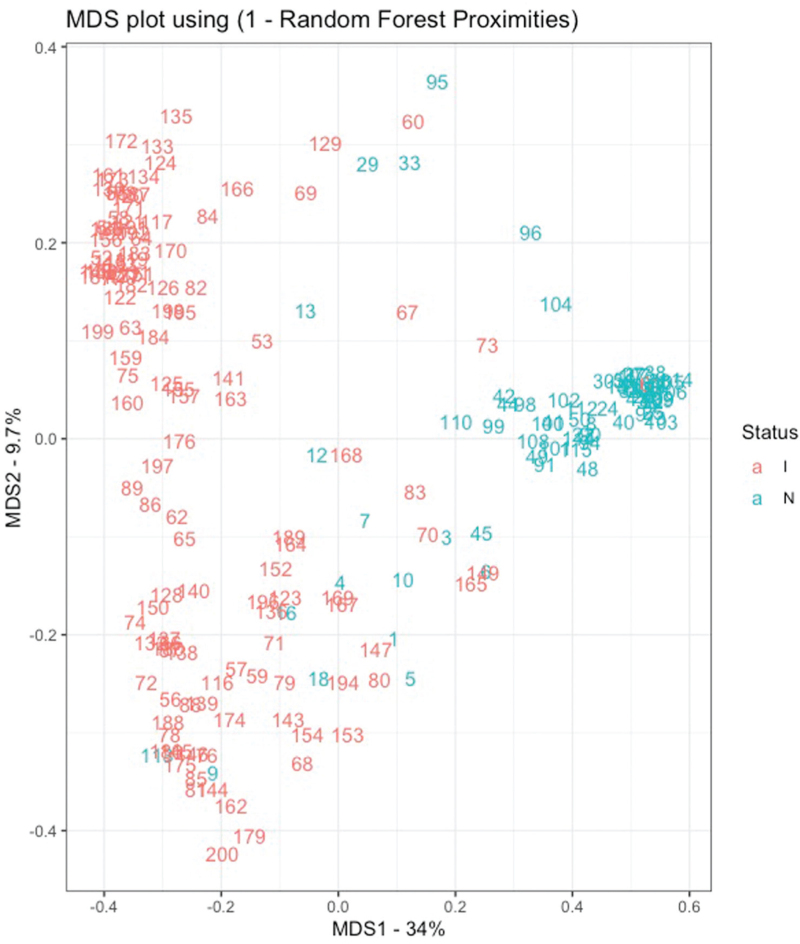
Hospitalised patient profiles with SARS-CoV2, 0 and their baseline findings.

In addition, we utilised a random forest model trained with 200 trees, trained on predictive values of biomarkers, lymphocyte subpopulations and immunoglobulins at admission, which evidenced an estimated out-of-the-box error of 8%. To display the results of the forest plot, we used a multidimensional scaling graph, revealing a clustering pattern among NON-ICU patients (Fig. 4).

Generation of profiles by multidimensional scaling analysis. Multidimensional statistical scaling was performed to visually cluster these cases according to (dis)similarity on the basis of their base line (within 0 to 48 h of a patient's admission) biochemical finding profiles, including lymphocyte subpopulations. ICU patients are shown in red, NON-ICU patients in blue. Large distances between individuals in this two-dimensional space indicate highly dissimilar baseline findings, while clustering of samples indicates similar baseline findings. The *x-*axis and y-axis represent the distance in percentage (%) from the random forest proximity matrix.

### Trend analysis

**Fig. 5:**
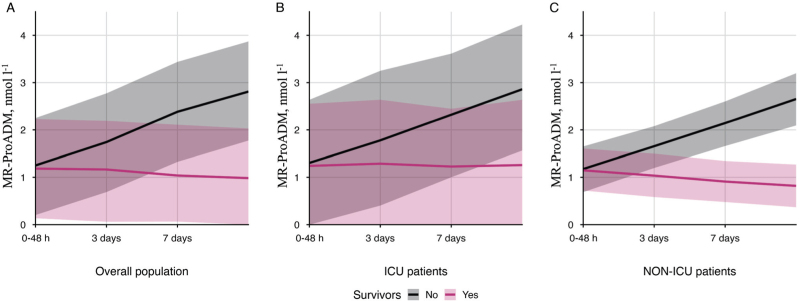
Representation of MR-ProADM values in surviving (in red) and nonsurviving (in black) patients within the overall population (panel A), the ICU population (panel B), and the NON-ICU population (panel C) at different timepoints (within 48 h of ward admission, day 3, day 7).

The analysis evaluating the time course of the values measured within 48 h to the admission, at day 3, and at day 7, revealed a statistically significant difference between survivors and nonsurvivors for MR-proADM values (*P* < 0.001), CD3+CD4+ values (*P* < 0.001), CD56+ values (*P* = 0.031). Details are shown in Table 5. When evaluating MR-ProADM values over time in both ICU and NON-ICU settings among survivors and nonsurvivors, a statistically significant difference (*P* < 0.001) was observed in both cohorts. The specific trend of MR-proADM values in nonsurvivors – both in ICU and non-ICU – is represented in Fig. 5.

**Table 5:** Trend over time of the mean values with corresponding 95% confidence intervals of midregional-proadrenomedullin in overall, ICU and NON-ICU patients at different timepoints (within 48 h of ward admission, day 3, day 7), stratified by survival status

	0 to 48 h		Day 3		Day 7		
	Survivors	Nonsurvivors	Survivors	Nonsurvivors	Survivors	Nonsurvivors	*P*
Overall population							
MR-ProADM (nmol l^−1^)	1.18 (2.23 to 0.13)	1.25 (2.25 to 0.20)	1.17 (2.19 to 0.06)	1.74 (2.77 to 0.69)	1.04 (2.11 to 0.06)	2.38 (3.44 to 1.33)	<0.001
ICU							
MR-ProADM (nmol l^−1^)	1.24 (2.55 to 0.00)	1.30 (2.64 to 0.00)	1.29 (2.64 to 0.00)	1.78 (3.25 to 0.40)	1.23 (2.45 to 0.00)	2.32 (3.61 to 1.00)	<0.001
NON-ICU							
MR-ProADM (nmol/l^−1^)	1.15 (1.61 to 0.72)	1.17 (1.65 to 0.69)	1.04 (1.50 to 0.58)	1.66 (2.08 to 1.20)	0.91 (1.34 to 0.48)	2.14 (2.60 to 1.66)	<0.001

ICU, intensive care unit; MR-proADM, midregional-proadrenomedullin.

We detected no bias in the selection of participants in the study, in classification of interventions, due to deviations from intended interventions, in measurement of outcomes and in selection of the reported result. The data reported had a missing data rate of less than 5%.

## Discussion

This study is the first to confirm the effectiveness of MR-proADM as a predictor of disease severity and patient mortality in both ICU and non-ICU COVID-19 patients. MR-proADM, in combination with clinical scores like SAPS II and MuLBSTA, enhances the prediction of mortality in non-ICU settings. Lymphocyte dysfunction, particularly T-lymphocyte and natural killer cell dysfunction, appears to contribute to COVID-19 severity, mainly in ICU patients. MR-proADM is a biomarker that reflects endothelial damage and correlates with disease severity in respiratory infections and sepsis.

The clinical relevance of MR-proADM, a biomarker of endothelial damage whose variations correlate with changes in vascular permeability, the inflammatory cascade, endothelial barrier regulation and microcirculation performance, has been steadily growing in recent years. In fact, its increase in plasma seems to correlate with disease severity both in case of respiratory infections and sepsis/septic shock and can be of clinical utility, especially in the emergency department.^29^ Recently, its predictive value has been highlighted in the context of COVID-19-related severe acute respiratory syndrome.^9^ However, until now, there has been a lack of data regarding MR-proADM in patients with different disease severity (from ordinary wards to ICU) and with repeated measurements over time.

In our cohort, considering the values at admission and the trend over the first 7 days, MR-proADM proved to have an adequate prognostic value in both areas of application (ICU and non-ICU patients), apparently showing a better prognostic performance than other ‘traditional’ inflammatory biomarker considered, such as CRP or procalcitonin, or other biomarkers used in COVID-19 patients, such as D-dimer, LDH and ferritin.^30^

The combination of a biomarker (e.g. MR-proADM) with a validated and commonly used clinical severity score, previously reported with the term ‘bio-scores’,^31^ is of particular clinical value, and the combination of MR-proADM with severity scores such as SAPS II and SOFA has provided encouraging results in septic patients.^32^ However, in previous experiences of using pro-ADM in COVID-19 patients,^17^ the combined use with specific severity scores had not been evaluated or had not given advantageous results in terms of effectiveness.

In the present study, the comparison of the different ROC curves shows better accuracy and AIC for the model, including clinical criticality predictivity scores, such as SAPS, SOFA and MuLBSTA, combined with MR-proADM measurement, as shown in Table 4.

The models also show that MR-proADM has good sensitivity (0.91) with low specificity in discriminating survivors from nonsurvivors, resulting in a PPV of 0.82 and an NPV of 0.72. Among the clinical severity scores, SAPS II would seem to have the highest sensitivity (0.94) and specificity (0.68), in discriminating survivors from nonsurvivors. However, it has to be considered that SAPS II has only been validated in the ICU population and it is rather complex to calculate in an emergency context when providing an assessment of the mortality risk that depends on the patient's presentation.

Despite the relatively low specificity, MR-proADM, as a highly specific biomarker of endothelial damage, seems to be able to provide indications about the probability of a patient's short-term evolution long before the clinical condition deteriorates and/or provide indications about the expected trend if evaluated repeatedly over time.^33^ Its use, therefore, appears particularly important in the initial and continuous evaluation of the COVID-19 patient at different levels of severity.

Based on the random forest analysis, we can validate the findings from our previous data and the existing literature. It is not the first time that a machine learning technique has been used to explore the behaviour of MR-proADM in hospitalised patients.^13^ In this case, we decided to use the random forest instead of the decision tree because our hypothesis was complex, with many variables and interactions among them, and to reduce overfitting. Our artificial intelligence model, trained on predictive values of biomarkers, lymphocyte subpopulations and immunoglobulins at admission, reveals a robust clustering pattern among NON-ICU-hospitalised patients. Furthermore, ICU-admitted patients exhibit distinct clustering within two well defined clusters, clearly differentiating them from the NON-ICU patients (Fig. 4). These results suggest that the comprehensive set of parameters examined holds promise as an initial approach for assessing patient severity upon admission to the medical ward.

### T-lymphocyte dysregulation among SARS-CoV2 population

The literature on COVID-19 immunology provides conflicting results regarding T-lymphocyte dysregulation and its impact on disease outcomes. Severe COVID-19 cases are often associated with elevated IL-6 and IL-10 levels and T lymphocytopenia, but the understanding of T-CD4 and T-CD8 functions in SARS-CoV-2 patients is limited.^34^ The relationship between the host immune status, including humoral and cellular responses, and disease outcomes remains partially evaluated with inconclusive results. Additionally, there is limited knowledge about immunoglobulin changes and specific subtypes in COVID-19.^35,36^

We have previously demonstrated, with a study on ICU critically ill COVID-19 patients, that lymphocyte subpopulations such as CD3, CD4, CD8 or the level of serum immunoglobulins (subgroups IgA, IgM and IgG) obtained within 48 h of admission or over time, were not statistically different between survivors and nonsurvivors. Therefore, no evidence emerged to support their use to predict disease severity and mortality.^17^

In the same direction, Mathew *et al.*^37^ proposed an immune profile identifying three primary immunotypes among COVID-19 patients: immunotype 1, associated with T-CD4 and T-CD8 hyperactivation, resulting in T-cell exhaustion and impaired Th-follicular cells alteration and leading to a higher level of severity; immunotype 2, with a lower activation of T-CD4 and T-CD8 and the presence of proliferating B-memory lymphocytes, which does not appear to be associated with high disease severity; immunotype 3, with a lack of activated B and T lymphocytes, negatively correlating with disease severity and providing protective effects against severe disease. The clinical applicability of this classification remains, however, uncertain. Additionally, Iannetta *et al.*^38^ found that early T-lymphocyte counts predict the severity of SARS-CoV2, whereas evidence for T-CD4 and T-CD8 subpopulations is weaker.

In this study, the results were not robust enough to confirm any of these findings. Although there were significant differences in T-lymphocyte subpopulations across different ward settings (Table 2b), multivariate analysis only weakly supported low levels of T lymphocytes, T-CD4 and T-CD8 as predictors of severity, primarily in ICU patients (Table 2b and Table 3). According to emerging literature, lymphocyte subpopulation depletion may be due to the increased severity of the disease caused by a higher level of host inflammation. However, a condition of immunosuppression due to the critical condition of the ICU patient further complicates the evaluation of this finding.^39^ Our data indicate a prognostic value for T lymphocytes (*P* = 0.03), T lymphocytes CD4 (*P* < 0.001), CD56 NK lymphocytes (*P* = 0.05) in the ICU group, and B-lymphocytes (*P* = 0.025) in the NON-ICU group (Table 2b). However, these findings should be interpreted with caution because of the different trends observed depending on the reference context. While T-lymphocyte dysfunction seems to play a role in defining COVID-19 severity in ICU populations, further data is needed to validate these observations.

Given the limited clinical value of immunological data and the highlighted limitations of traditional biomarkers, the importance of MR-proADM as a versatile biomarker should be emphasised. Its validity holds regardless of the ward type and in relation to both admission values and trends. Furthermore, combining MR-proADM with traditional severity scores, such as SAPS II, at admission, can help clinicians determine the severity and appropriate setting for each patient.

### Limitations

This study has certain limitations. The sample size, although larger than previous studies, remains limited. Additionally, the study period covered different pandemic waves with variations in logistical and therapeutic approaches, such as the use of steroids. The role of potential confounders, including co-infections, super-infections, renal dysfunction, myocardial alterations and immunological therapies, was not considered. Furthermore, the study focused solely on COVID-19 patients, and its findings cannot be generalised to other clinical contexts.

## Conclusion

MR-proADM admission values and their trends over time seem to be a suitable marker of COVID-19 patients’ severity both in and outside the ICU, with a better ability to predict mortality than any other considered biomarker. The combination of this biomarker with a validated and commonly used clinical severity score, such as SAPS II and MuLBSTA, appears to improve its sensitivity, specificity, PPV and NPV in predicting in-ward mortality with a relevant clinical relapse. T-lymphocyte dysfunction appears to play a role in defining the severity of COVID-19 in ICU populations, pending more consistent data.

## Supplementary Material

Supplemental Digital Content
