# Supplementary material for: Critical and non-critical coronavirus disease 2019 patients: which is the most predictive biomarker for disease severity and outcome? A multicentre prospective cohort study comparing mid-regional pro-adrenomedullin, inflammatory and immunological patterns
Source: Eur J Anaesthesiol Intensive Care. 2023 Nov 21;2(6):e0039. doi: 10.1097/EA9.0000000000000039 (PMC11798374; doi:10.1097/EA9.0000000000000039)

**Supplementary material**

**Critical and non-critical COVID-19 patients: which is the most effective biomarker? A multicenter prospective observational study comparing mid-regional pro-adrenomedullin, inflammatory and immunological patterns.**

Montrucchio G, Balzani E, Sales G, Bolla C, Della Selva A, Perotto M, Pomero F, Ravera E, Rumbolo F, Fanelli V, Mengozzi G, Brazzi L

Supplementary – e-Table1. Significance (*P*) on univariate analysis, in the overall patient population and in both ICU and NON-ICU patients: grouped by survivors and non survivors.

| **Descriptive data used** | **ICU (*P*)**  **survivors vs non survivors** | **NON-ICU (*P*)**  **survivors vs non survivors** | **Overall (*P*)**  **survivors vs non survivors** |
| --- | --- | --- | --- |
| Age, mean ± SD | 0.07467 | 0.2774 | 0.445 |
| BMI, median [IQR] | 0.4153 | 0.07711 | 0.3083 |
| Male, n (%) | 0.1164 | 0.9902 | **0.04091** |
| SOFA score, at admission, median [IQR] | 0.1675 | 0.6636 | **< 0.001** |
| MuLBSTA score, at admission, median [IQR] | **< 0.001** | 0.1499 | **< 0.001** |
| SAPS II score, at admission, median [IQR] | **< 0.001** | 0.07333 | **< 0.001** |

eFigure 1. Consort flow diagram.


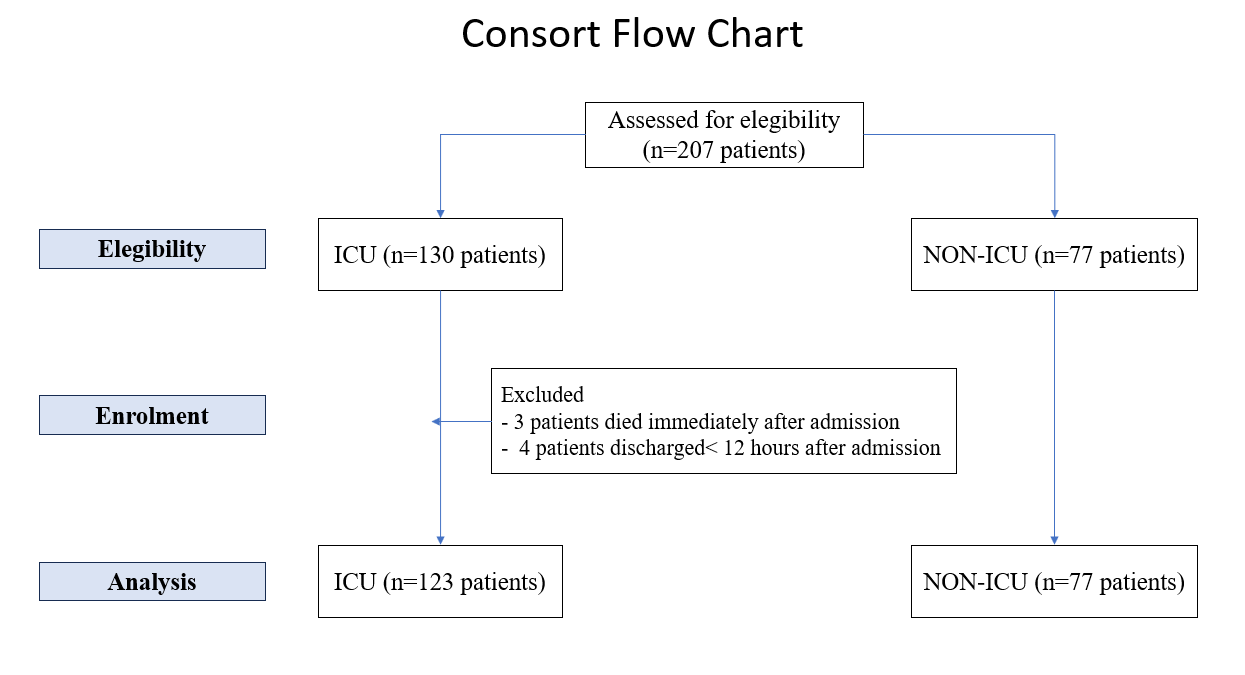


eFigure 2. Receiver Operating Characteristic (ROC) curve for MR-proADM and clinical scores. The ROC curve plots the true positive rate (sensitivity) against the false positive rate (1-specificity) at various threshold settings. The area under the curve for MR-proADM (AUC) is 0.88, indicating good discriminatory power of the mortality.


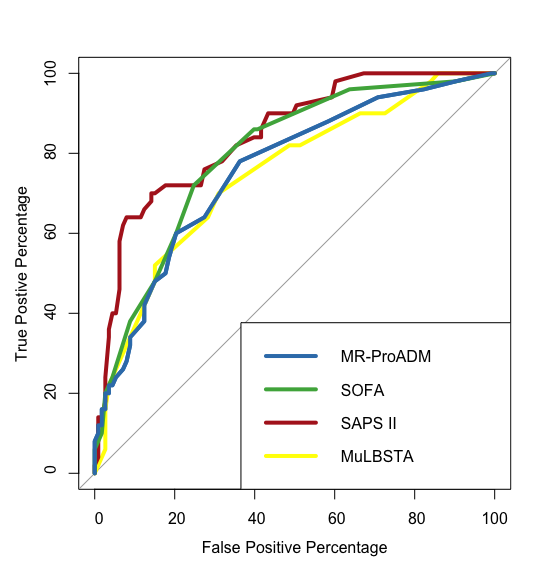

Supplement: Supplemental Digital Content [file ejaic-2-e0039-s001.docx]
